# Rapid fabrication of anti-corrosion and self-healing superhydrophobic aluminum surfaces through environmentally friendly femtosecond laser processing

**DOI:** 10.1364/OE.400804

**Published:** 2020-11-10

**Authors:** Gan Yuan, Yu Liu, Chi-Vinh Ngo, Chunlei Guo

**Affiliations:** 1 The Photonics Laboratory, State Key Laboratory of Applied Optics, Changchun Institute of Optics, Fine Mechanics and Physics, Chinese Academy of Sciences, Changchun, Jilin 130033, China; 2 University of Chinese Academy of Science, Beijing 100049, China; 3 The Institute of Optics, University of Rochester, Rochester, New York 14637, USA; 4 chivinh@ciomp.ac.cn; 5 guo@optics.rochester.edu

## Abstract

The development of superhydrophobic metals has found many applications such as self-cleaning, anti-corrosion, anti-icing, and water transportation. Recently, femtosecond laser has been used to create nano/microstructures and wetting property changes. However, for some of the most common metals, such as aluminum, a relatively long aging process is required to obtain stable hydrophobicity. In this work, we introduce a combination of femtosecond laser ablation and heat treatment post-process, without using any harsh chemicals. We turn aluminum superhydrophobic within 30 minutes of heat treatment following femtosecond laser processing, and this is significantly shorter compared to conventional aging process of laser-ablated aluminum. The superhydrophobic surfaces maintain high contact angles greater than 160° and low sliding angles smaller than 5° over two months after the heat treatment. Moreover, the samples exhibit strong superhydrophobicity for various types of liquids (milk, coffee, CuPc, R6G, HCl, NaOH and CuCl_2_). The samples also show excellent self-healing and anti-corrosion properties. The mechanism for fast wettability conversion time is discussed. Our technique is a rapid process, reproducible, feasible for large-area fabrication, and environment-friendly.

## 1. Introduction

The harsh and moisture environment can accelerate corrosion for metals. Superhydrophobic surfaces, where water cannot stay on the surface, usually possess anti-corrosion effect. Normally, hydrophobicity is defined when the contact angle of a water droplet on the surface is great than 90°; and superhydrophobicity is defined when the contact angle is greater than 150° with a sliding angle smaller than 10° [1,2]. The superhydrophobic property of lotus leaf is due to nano/microstructures on its surface and the low surface energy [3]. These two factors can also be applied to other materials to make superhydrophobic surfaces for dealing with corrosion problems.

A variety of methods have been used to make nano/microstructures, such as Chemical Vapor Deposition (CVD), electrochemical corrosion, plasma sprayed, and other chemical-based methods [4–8]. However, these techniques are costly and complex. With the development of laser techniques, many researchers have focused on femtosecond laser processing to make nano/microstructures on different materials, which is lower cost and faster fabrication speed than CVD and other chemical-based methods [9,10]. Femtosecond laser processed surfaces also show drastic changes in surface wettability [11–13]. Stable hydrophobicity may be more difficult to obtain for some metals, such as aluminum. Often time, an aging process or chemical coating is applied following laser treatment. For example, aluminum requires 14–30 days to convert from superhydrophilicity to superhydrophobicity [14–17]. Another approach is coating chemicals such as Teflon, lauric acid or other harsh chemicals, on the femtosecond-laser-ablated metal to reduce the surface energy without waiting for any aging time [18–20]. However, this approach requires a few hours to over one day for the coating process. In several techniques, complex coating system or harsh chemicals are required. In our previous researches [21,22], we have tried to combine nanosecond laser and low temperature annealing process to accelerate the wettability conversion time from superhydrophilic to superhydrophobic. However, the required annealing treatment time is at least 6 hours to get the good superhydrophobicity with a high contact angle (greater than 150°) and a low sliding angle (smaller than 10°). Moreover, the self-healing property and anti-corrosion property have not been studied on these researches.

In this research, a combination with femtosecond laser ablation and heat treatment post-process is introduced. The femtosecond laser is employed because it has a different interaction mechanism between the laser beam and metal surface, compared to that of the nanosecond laser [23–25], which can affect wettability conversion time. The proposed technique can solve the current limitations of other conventional techniques; and this technique also improves significantly the fabrication time as well as the superhydrophobic property compared to our obtained results in the previous works. First, a femtosecond laser was used to ablate a grid pattern on a flat aluminum surface to create nano/microstructures. Then, the just-after-laser-ablated surfaces were put in a commercial oven at 200 °C for heat treatment post-process. Interestingly, within 30 minutes under heat treatment, the samples converted from superhydrophilic to superhydrophobic, without the usage of any harsh chemicals. The wettability conversion time of femtosecond-laser-ablated aluminum can be shortened significantly from approximately 30 days in the common aging process to 30 minutes in this treatment post-process. The fabrication time is shortened approximately 1440 times. Compared to our previous studies, the wettability conversion time is reduced roughly from 6 hours to 30 minutes, which is shortened 12 times. Moreover, the superhydrophobic property in this technique shows better performance compared to our previous results. All the samples exhibited contact angles greater than 155° and sliding angles smaller than 5°. In this research, the fabricated superhydrophobic aluminum surface showed excellent effects such as stable superhydrophobicity (over two months), varied liquid-repellency, anti-corrosion, and self-healing. Moreover, the proposed technique demonstrated its reproductivity and feasibility of large-area fabrication.

## 2. Experimental

### 2.1 Material

Pure aluminum plate (99.999% purity, Zhong nuo, China) with 1 mm thickness was employed as the substrate. Before femtosecond laser ablation, the plate was divided into small pieces of 10 mm x 10 mm and small pieces of 20 mm x 20 mm. The small pieces were cleaned by ultrasonic with ethanol in 10 minutes, then cleaned by deionized (DI) water for 10 minutes to remove any initial contaminants on the aluminum surface.

### 2.2 Fabrication method

**Fig. 1.**
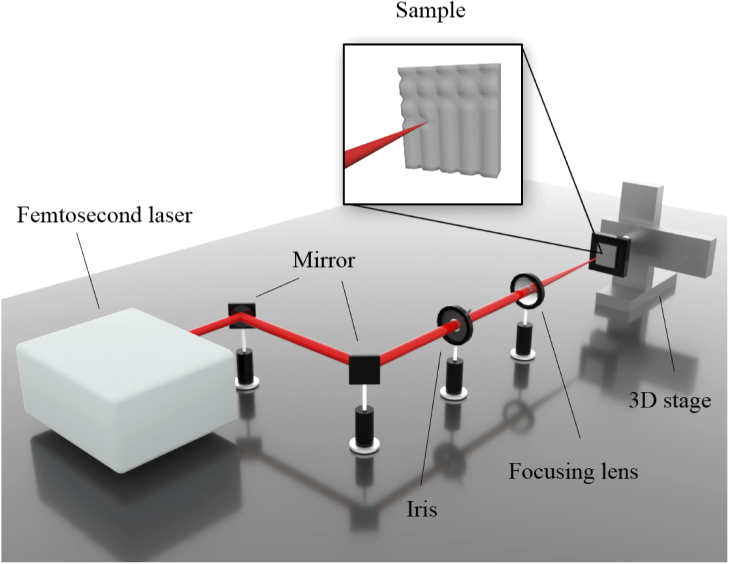
The schematic diagram of the femtosecond laser system.

Figure 1 represented the schematic diagram of the femtosecond laser system. A central wavelength of 800 nm laser was generated by Ti: sapphire femtosecond laser amplifier system (Spitfire, Spectra-physics Inc.), with horizontally polarized 40 fs Gaussian pulses and 1 kHz repetition rate. The laser arrived at the 200 mm focal length lens by five times of reflection. The focus diameter on the aluminum surface was about 85 μm. Samples were fixed on a 3-dimensional translation stage. The laser of 200 mW power was used to create grid patterns on aluminum with different step sizes (100 μm, 300 μm, and 500 μm). Five samples were made for each step size. The focusing point of the femtosecond laser was scanned with 1 mm/s in two directions (x, y) perpendicular to the sample surface by the movement of the 3-dimensional stage. Before heating the femtosecond-laser-ablated samples, a mechanical pump was used to remove the existent gas in the commercial oven and then let new fresh air come inside. Then, the samples were put inside the oven at 200 °C for 30 minutes for heat treatment.

### 2.3 Measurement method

Scanning electron microscopy (SEM: Hitachi S-4800) and confocal microscopy (KEYENCE VK-X200) were used to measure the morphology of the aluminum surface. X-ray diffraction (XRD: BRUKER D8) was used for surface chemistry analysis, while energy-dispersive X-ray spectroscopy (EDS: Phenom Pro-X) could characterize the element on the aluminum surface and Fourier transform infrared (FTIR: Agilent technologies Cary 630) showed organic groups on surfaces. For the measurement of contact angle and sliding angle, a 10 μl water droplet was used. In order to investigate the corrosion property of the treated samples, 5 wt % copper (II) chloride (CuCl_2_) solution was used. In this experiment, a flat surface without laser ablation and a just-after-laser-ablated aluminum surface were used to compare the anti-corrosion property with a superhydrophobic aluminum surface using femtosecond laser ablation and heat treatment. In addition, electrochemical measurements were performed on the superhydrophobic aluminum surfaces by an electrochemical workstation (EC-Lab VMP3) in 3.5 wt % NaCl solution at room temperature. The measurements were conducted in a three electrodes cell with an Ag/AgCl reference electrode and a platinum mesh counter electrode. The sample with an exposed area of 1 cm^2^ circle serves as the working electrode. Before the electrochemical measurements, all the samples were immersed in the 3.5 wt % NaCl solution for 60 minutes to obtain a stable open circuit potential (OCP). Polarization curves were obtained for untreated and superhydrophobic aluminum surfaces by using a scan rate of 1 mV/s in the range of ±100 mV versus the OCP.

## 3. Results

### 3.1 Surface morphology

**Fig. 2.**
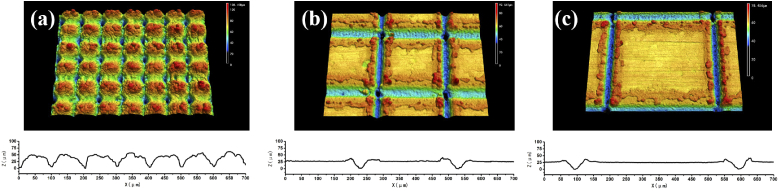
3D confocal microscope images of the femtosecond-laser-ablated aluminum surfaces with different step sizes of: (a) 100 μm, (b) 300 μm, and (c) 500 μm. The section height of each step sizes was shown on the bottom of each figure.

Figure 2 showed the 3D confocal microscope images of the aluminum surface with different step sizes after the femtosecond laser ablation. For the 100 μm step size, the micro-valleys with nanostructures were made by the femtosecond laser ablation. The height of the micro-valleys was approximately 50 μm. When increasing the step size to 300 μm or 500 μm, the ablation lines with the deposited aluminum debris along two sides of these lines can be seen on the surface of the substrate.

**Fig. 3.**
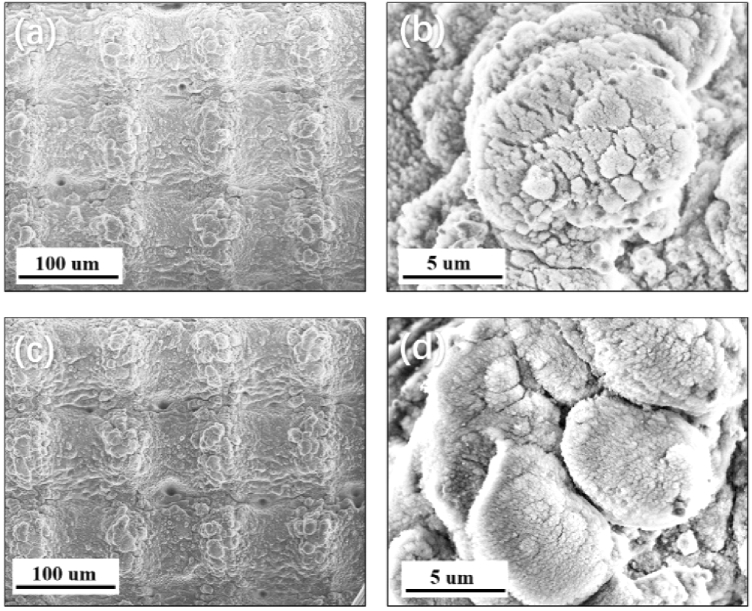
SEM images of: (a), (b) the just-after-femtosecond-laser-ablated aluminum surface, and (c), (d) the femtosecond-laser-ablated aluminum surface after heat treatment with the step size of 100 μm.

When the femtosecond laser ablated on the aluminum surface to make the lines, many ablated nano-particles and micro-particles were produced. These particles were re-deposited along two sides of the ablated lines. Figures 3(a) and 3(c) showed the bulges of the surface with the scale as 100 μm, approximately. On the top areas as shown in Figs. 3(b) and 3(d), many nanoscale particles appeared on the surface of the bulges. After laser ablation, the nano/microstructures appeared. However, the shapes and structures before and after heat treatment post-process did not change. Therefore, since femtosecond laser ablation, the nano/microstructures had not changed over time.

### 3.2 Surface chemistry composition

Three main elements were detected on the aluminum surfaces with different step sizes as shown in Table 1. After femtosecond laser ablation, the oxygen content had an extremely increment from approximately 10% to 40–55%. This could come from oxidation of aluminum during the interaction with the femtosecond laser beam. Additionally, the carbon content increased after laser ablation. Before the heat treatment, the ratio between aluminum and carbon content (Al/C) is approximately 3.61, 3.38, and 3.29 at the step size of 100 μm, 300 μm, and 500 μm, respectively. This indicated the amount of aluminum content was much more than the amount of carbon content. However, after the heat treatment, the Al/C ratios were changed as 0.92, 0.84, and 1.03 at the step size of 100 μm, 300 μm, and 500 μm, respectively. This demonstrated that the carbon content on the laser-ablated surface after the heat treatment increased incredibly, even the amounts of carbon content were greater than that of aluminum content in the case of 100 μm and 300 μm step size. In this experiment, the change of step sizes did not affect the amount of carbon content. For example, before the heat treatment, the carbon contents were approximately 11.2%, 11.47%, and 10.87% at the step size of 100 μm, 300 μm, and 500 μm, respectively. Similarly, after the heat treatment, the carbon contents were similar for these step sizes, approximately 30%. Therefore, after femtosecond laser ablation, oxygen content increased, and this composition was nearly maintained after heat treatment post-process. The carbon content increased after femtosecond laser ablation, and especially, the content increased three times after heat treatment. The increment of carbon content after heat treatment should be investigated more by other surface analysis techniques.

**Table 1.** The EDS results of the samples with different step sizes and conditions

Conditions	Before heat treatment			After heat treatment			Flat Surface
Step size(μm)	100	300	500	100	300	500	
**Al (%)**	40.40	38.81	35.77	26.8	27.6	30.35	90.39
**O (%)**	48.39	49.72	53.36	44.11	39.66	40.07	9.61
**C (%)**	11.20	11.47	10.87	29.09	32.73	29.58	/
**Al/O**	0.83	0.78	0.67	0.61	0.70	0.76	9.41
**Al/C**	3.61	3.38	3.29	0.92	0.84	1.03	/
**O/C**	4.32	4.33	4.91	1.52	1.21	1.35	/

**Fig. 4.**
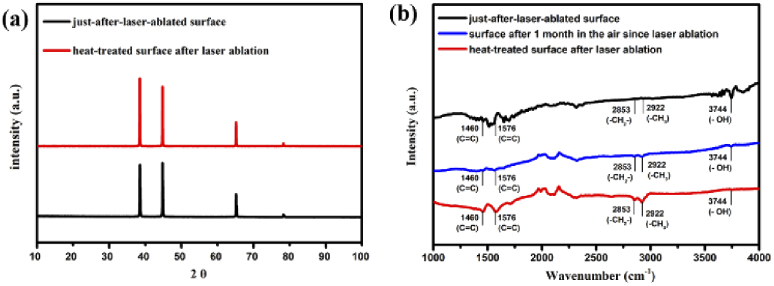
(a) The XRD results of the just-after-laser-ablated surface and the heat-treated surface after laser ablation. (b) The FTIR results of the just-after-laser-ablated surface, the surface after 1 month in the air since laser ablation and the heat-treated surface after laser ablation.

The crystalline structures laser-ablated aluminum surface before and after heat treatment were compared as shown in Fig. 4(a). The peaks of aluminum were observed clearly. Four diffraction peaks of Al surfaces at 2θ = 38.46°, 44.7°, 65.08°, and 78.22° are assigned to Al (111), Al (200), Al (220), and Al (311) respectively, in agreement with JCPDS Card No. 04-0787. New crystalline structures did not form after the heat treatment post-process. FTIR was employed to investigate the bonding of the aluminum surface, as shown in Fig. 4(b). The results of the flat aluminum surface and on the just-after-laser-ablated surface were similar. However, after heat treatment post-process, new peaks appeared clearly at 1460 cm^-1^ as –C = C–, at 1576 cm^-1^ as –C = C–, at 2853 cm^-1^ as –CH_2_– and at 2922 cm^-1^ as –CH_3_. This demonstrated that the organic adsorption could happen. Additionally, the organic adsorption peaks at 2853 cm^-1^ (–CH_2_–) and 2922 cm^-1^ (–CH_3_) were also found on the femtosecond-laser-ablated aluminum surface, which had put in the ambient air for one month since laser ablation (without any heat treatment post-process). The intensities of these two peaks were smaller than the intensities of two peaks, which were found on the heat-treated surface after laser ablation. The heat treatment post-process might play a role as an acceleration post-process.

### 3.3 Surface wettability

**Fig. 5.**
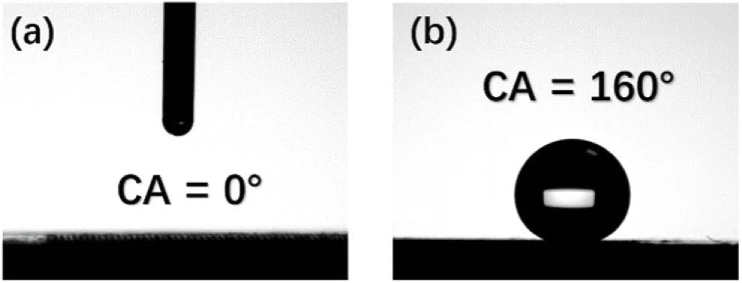
The contact angles of: (a) the just-after-laser-ablated aluminum surface of 100 μm step size, and (b) the heat-treated aluminum surface after laser ablation of 100 μm step size.

The contact angle of the flat aluminum surface was 49°, i.e. hydrophilicity. After femtosecond laser ablation, the contact angle became 0°, i.e. superhydrophilicity. The water droplet diffused suddenly on the just-after-laser-ablated surface to exhibit the contact angle of 0° as shown in Fig. 5(a). When heating the femtosecond-laser-ablated samples at 200 °C for 30 minutes, the contact angle increased to approximately 160° as shown in Fig. 5(b). The sliding angles of the heat-treated surface after laser ablation surfaces were smaller than 5°. Therefore, after heat treatment, the just-after-laser-ablated surface converted from superhydrophilicity to superhydrophobicity.

**Fig. 6.**
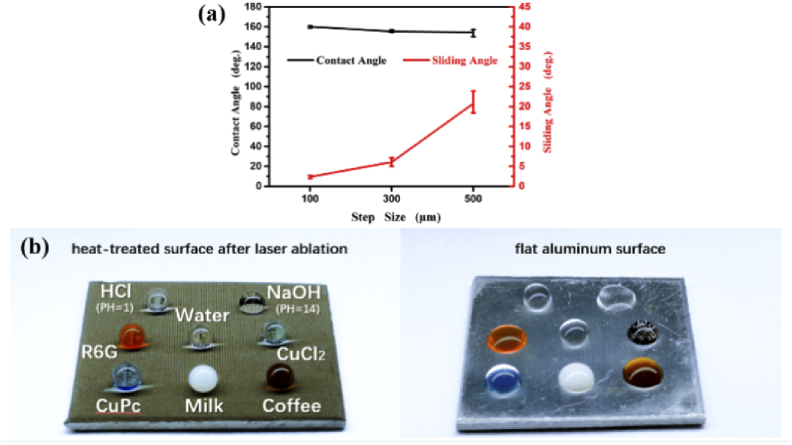
(a) The contact angles and sliding angles of the heat-treated aluminum surface after laser ablation with different step sizes of 100 μm, 300 μm, and 500 μm; and (b) liquid repellency with various types of liquids on the heat-treated surface.

The effect of step size on wettability conversion was shown in Fig. 6(a). After heat treatment, the femtosecond-laser-ablated aluminum surfaces with different step sizes of 100 μm, 300 μm, and 500 μm had all high contact angles greater than 150°. The contact angles decreased a bit with the increment of the step size. Each value as shown in Fig. 6 was the average value of measuring contact angles of the three samples, and the error bars were the maximum and the minimum values of the measurement. When the step size increased, the sliding angles also increased from the values smaller than 5° at 100 μm step size to the values approximately 5° at 300 μm step size and then to the values approximately 20° at 500 μm step size. In this study, the 100 μm step size showed the best performance of superhydrophobic due to the largest contact angles and the smallest sliding angles, compared to the results of 300 μm and 500 μm step size. After heat treatment, all samples with different step sizes in this research exhibited superhydrophobic property. The samples were stored in the room and were re-measured the contact angles and the sliding angles after two months. All samples demonstrated the stability of superhydrophobicity with the error bar of contact angles smaller than 3°, as shown in Supplement 1, Table S1. Moreover, the superhydrophobic aluminum surface showed the repellent property to various types of liquids such as milk, coffee, copper (II) chloride (CuCl_2_), rhodamine 6G (R6G), copper (II) phthalocyanine (CuPc), and even hydrochloric acid (HCl with pH=1), sodium hydroxide (NaOH with pH=14), compared to the flat aluminum surface, as shown in Fig. 6(b).

## 4. Discussions

### 4.1 Mechanism for femtosecond laser the wettability conversion

When combining femtosecond laser ablation and heat treatment post-process, the morphology and the chemical composition of the aluminum surfaces were changed, which could reveal the mechanism for the fast wettability conversion from superhydrophilicity to superhydrophobicity. Due to the short time scales involved in the ablation of the femtosecond laser, the ablation process can be considered as a direct solid-vapor (or solid-plasma) conversion, which is different from the nanosecond laser ablation process; i.e. the energy first heats the solid target surface to the melting point and then to the vaporization temperature. This is the great advantage of femtosecond lasers compared to nanosecond lasers because of the dissipation of energy or the minimization of the heated affected zone. With the unique solid-vapor conversion, femtosecond laser ablation could create micro/nano structures as well as produce many nanoparticles on the surface, which increased the area that adsorbs organic matter in the air. In addition, the femtosecond laser directly evaporated the aluminum to make lotus-leaf-like structures surface as shown in Fig. 2(a) and Fig. 3; whereas, this could not be made by nanosecond laser. The nanosecond laser made burrs, as microstructures with double sharp tent shape, along the ablated lines by melting process [23–25]. L. Jiang et al. found that lotus-leaf-like structures can decrease the content area with the water and increase the superhydrophobic property [1]. In addition to the change of surface morphology, the chemical composition of the aluminum surface also changed when interacting with femtosecond lasers. When the femtosecond laser beam interacts with the aluminum surface, it can reach a high temperature up to approximately 5447 °C [26], which can easily oxidize the metal surface [27]. Aluminum and aluminum oxide are both inherently hydrophilic, which is strongly attracted to water. Therefore, when the surface roughness increased, the femtosecond-laser-ablated aluminum surface exhibited superhydrophilic property. This property was followed by Wenzel’s theory [28].

Other researchers have found that the femtosecond-laser-ablated aluminum surfaces convert from superhydrophilic to superhydrophobic when putting in the ambient air for a couple of weeks to a couple of months [14–17]. In this research, the femtosecond-laser-ablated samples, which were put in the ambient air for one month since laser ablation (without any heat treatment post-process), also converted from superhydrophilic to near superhydrophobic or superhydrophobic (135°—150°). The reason came from an organic adsorption mechanism. S. Banerjee has found that aluminum oxides, which were made at around 500 °C temperature by evaporation of the initial water layer on the oxides, exist nanoholes. These nanoholes have adsorption capacity, which is always used to made alumina adsorbent balls with nanostructures and microstructures [29]. Similarly, in this research, the just-after-laser-ablated surface also had many aluminum oxide nanostructures compared to our previous study in nanosecond laser [21,22]. Therefore, the nanostructures acting like the alumina adsorbent may have higher adsorption capacity on the femtosecond laser treated surface compared to nanosecond laser treated one. Moreover, the hydroxyl group (–OH) is also an effective adsorptive site, could adsorb the organic matters such as acetic acids, formic and polymeric hydrocarbons from air moisture [30,31]. Therefore, the organic matter in the ambient air are adsorbed on the femtosecond-laser-ablated surface to reduce the surface energy. The FTIR result on the femtosecond-laser-ablated samples, which were put in the ambient air for one month since laser ablation (without any heat treatment post-process), demonstrated the appearance of hydrophobic (–CH_2_–) bonding, (–CH_3_) bonding, and (–C = C–) [32]. The increment of the surface roughness and the appearance of hydrophobic bonding makes the femtosecond-laser-ablated aluminum samples, which were put in the ambient air for one month since laser ablation (without any heat treatment post-process), became superhydrophobic. One month is a relatively long time and is not suitable for any manufacturing purposes. Therefore, by applying the heat treatment post-process for 30 minutes at 200 °C, the femtosecond-laser-ablated surface showed high contact angles greater than 160° and low sliding angles smaller than 5°. The heat treatment post-process only played a role as an acceleration process of the inherent organic adsorption phenomenon on femtosecond-laser-ablated surfaces. After the heat treatment, the chemical compositions changed, compared to the results of the just-after-laser-ablated surfaces. The EDS results demonstrated the incredible increment of carbon content. The FTIR results also showed the clear appearance of (–CH_2_–) bonding and (–CH_3_) bonding, which were similar to the case of the femtosecond-laser-ablated aluminum samples, which were put in the ambient air for one month since laser ablation (without any heat treatment post-process). However, in the case of heat treatment, hydrophobic (–CH_2_–) bonding and (–CH_3_) bonding appeared with higher intensity. This agreed with the wettability results (contact angle and sliding angle). The femtosecond-laser-ablated surfaces after one month in the air (without any heat treatment post-process), which existed low intensity of hydrophobic bonding, showed high contact angle (135° – 150°) and no sliding angle; whereas, the heat-treated one (after heat treatment for 30 minutes), which existed high intensity of hydrophobic bonding, showed high contact angle (about 160°) and low sliding angle (smaller than 5°). Therefore, the heat treatment could support the femtosecond-laser-ablated aluminum to get sufficient organic matters to decrease its surface energy to show the superhydrophobic property. This acceleration process can reduce the common wettability conversion time to superhydrophobicity on the femtosecond-laser-ablated aluminum surface from one month to 30 minutes.

**Fig. 7.**
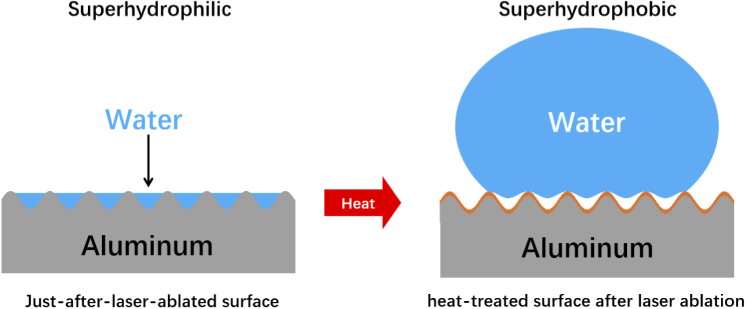
The mechanism for wettability conversion from superhydrophilicity to superhydrophobicity.

In summary, the wettability conversion from superhydrophilic to superhydrophobic aluminum surface depended on two main factors: surface roughness (nano/microstructures) and low surface energy of the materials. After femtosecond laser ablation, the aluminum increased its surface roughness with nanostructures and microstructures, without any formation of burrs or debris as same as the case of nanosecond laser ablation. These structures made by femtosecond are more absorbent for the organic matter in the ambient air. The heat treatment post-process accelerated the organic adsorption phenomenon on the femtosecond-laser-ablated aluminum surface to form an organic layer as shown in Fig. 7. The combination of nano/microstructures and hydrophobic organic layer makes surfaces superhydrophobic.

### 4.2 Reproductivity and feasibility for large-scale fabrication

**Fig. 8.**
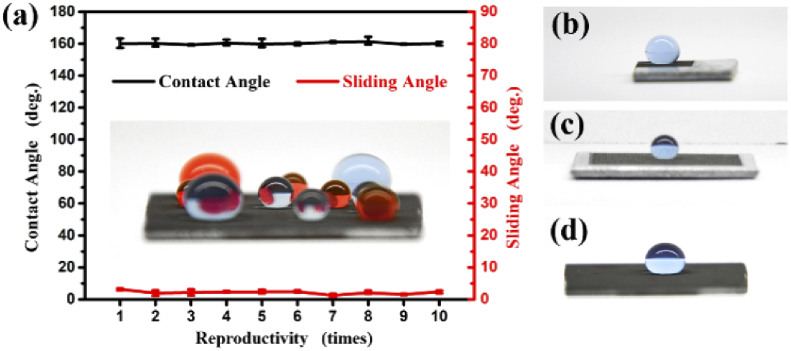
The contact angles and sliding angles of the heat-treated aluminum surface after laser ablation for the 10-time repetition.

The step size of 100 μm showed the best superhydrophobic performance among the obtained result in this research study. To investigate the reproductivity of this technique (femtosecond laser ablation and heat treatment), ten fabrication times were carried out with the same laser fabrication parameters. As shown in Fig. 8(a) the wettability results of 10-time repetitions were presented and each time includes three aluminum samples. All samples exhibited contact angle as approximately 160° and sliding angles as smaller than 5°. This demonstrated the reliability and reproductivity of the fabrication technique in this research. Moreover, all samples maintained their superhydrophobicity even over five months.

5 mm x 5 mm grid pattern area is small for any practical applications. Therefore, an investigation on the feasibility of large-scale fabrication was performed. Several samples were ablated to form the grid pattern by femtosecond laser with larger fabrication areas. Herein, samples with 15 mm x 15 mm (scale-up of 3 times) and samples with 20 mm x 20 mm (scale-up of 4 times) was employed. After laser fabrication, all samples were put in the oven for 30 minutes at 200 °C as same as the heat treatment conditions on the samples with the 5 mm x 5 mm grid pattern area. Interestingly, all samples showed the superhydrophobic property as shown in Figs. 8(b), (c), and (d). The contact angle and sliding angle of the 20 mm x 20 mm sample were similar to that of the 5 mm x 5 mm sample, as shown in Supplement 1, Fig. S1. The heat treatment time and the heat treatment temperature were independent of the fabrication area. This can be extremely useful when making a large-scale superhydrophobic aluminum surface for practical applications in the industry because the heat treatment time is short. This fabrication method can also be used to process different common metal surfaces into superhydrophobic showed in Supplement 1, Fig. S2. Visualization 1 showed the water bouncing on the large-scale heat-treated surface after laser ablation.

### 4.3 Self-healing property

**Fig. 9.**
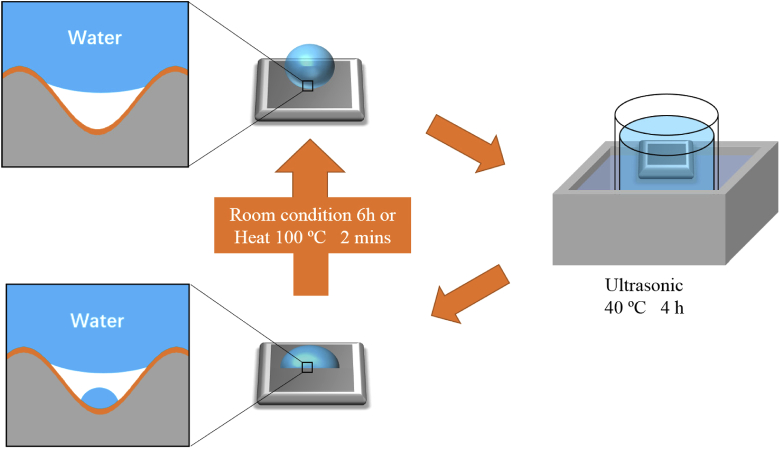
Schematic diagram of self-healing property.

The self-healing property of the obtained superhydrophobic aluminum surface was investigated by using ultrasonic cleaning. When the obtained superhydrophobic samples were cleaned under ultrasonic at 40 °C for 4 hours, they became hydrophobic and hydrophilic in different positions. After ultrasonic cleaning, samples were put in the room condition for 6 hours. Interestingly, they recovered their superhydrophobicity, called as self-healing effect. To reduce six hours of waiting time, other samples after ultrasonic cleaning were put on the hot plate at 100 °C for 2 minutes. They could also recover their superhydrophobicity. In this experiment, ultrasonic cleaning could make the water go inside of the channels between the nano/microstructures because of the vibration [33]. The air gap between the water and the superhydrophobic surface was removed. The water went through the gap between the nano/microstructures and formed a thin water layer inner the structures. When dropping water on these surfaces during contact angle measurement, the water layer can combine with the dropping water and make samples hydrophobic or hydrophilic. However, when the samples were put in the air for 6 hours, the thin water layer can be naturally evaporated. Therefore, the samples become superhydrophobic again. When samples were put on the hot plate, high temperatures at 100 °C can enhance the evaporation process of the resultant thin water layer from ultrasonic cleaning. The fabricated aluminum surfaces in this research can recover their repellent property after losing the superhydrophobicity, like the self-healing effect as shown in Fig. 9.

### 4.4. Anti-corrosion property

**Fig. 10.**
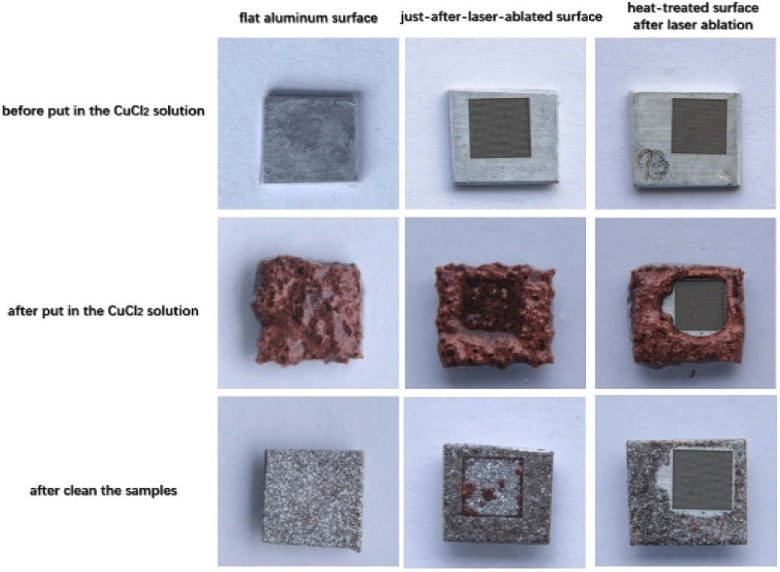
The pictures of the heat-treated surface after laser ablation, flat aluminum surface, and just after-laser-ablated surface before and after immersing into the copper (II) chloride CuCl_2_ for 3 minutes.

After putting the samples into the 5 wt % copper (II) chloride solution for 3 minutes, the red copper appeared on the whole surface of the flat surface and the just-after-laser-ablated aluminum surface; whereas the superhydrophobic aluminum surface did not appear the red color on the fabricated area, as shown in Fig. 10. This could demonstrate that there is no copper formation in this area. The area around this fabricated area of the superhydrophobic surface appeared red copper because this is the flat aluminum surface. It means that the sample after femtosecond laser and heat treatment exists the anti-corrosion property. Additionally, the whole corrosion process on the flat aluminum surface, on the just-after-laser-ablated aluminum surface, and on the superhydrophobic aluminum surface, were presented in Visualization 2. In the superhydrophobic area, the organic layer on the aluminum surface can protect the surface and it can also make an air barrier which prevents the solution to go inside the surface to contact aluminum. After immersing in the copper (II) chloride solution for 3 minutes, all the surfaces were ultrasonically cleaned. The taken images as shown in Fig. 10 indicated clear damage of the flat surface and the just-after-laser-ablated aluminum surface; whereas the superhydrophobic aluminum surface did not show any damage. The SEM images and the 3D structures, as shown in Supplement 1, Figs. S3 and S4, respectively, could support well for the anti-corrosion property of the superhydrophobic aluminum surface. While the strong damage of structures on the flat surface and the just-after-laser-ablated aluminum surface was observed clearly; the structures on the superhydrophobic aluminum surface were similar to the superhydrophobic ones before the anti-corrosion testing. Moreover, on the superhydrophobic aluminum surface after immersing into the 5 wt % copper (II) chloride solution, the contact angle almost did not change and the sliding angle increased a bit, approximately to 5°. The mechanism can briefly be explained by the reactions as shown as Eq. (1), (2), and (3). First, Cu^2+^ could replace the aluminum on the surface and became copper (Cu), which can be deposited on the surface of the sample. After this reaction, the corroded surface would turn to red because of the appearance of copper on the top of the sample surface. Then, the H^+^, which had been produced by hydrolyzed of Cu^2+^ and Al^3+^, could create hydrogen with the reaction of aluminum. During this reaction, bubbles could be formed on the aluminum surface. The superhydrophobic surface can prevent the corrosive solution to interact with the surface due to the air gap. The anti-corrosion property with 5 wt % copper (II) chloride on the superhydrophobic aluminum surface is demonstrated.

(1) $$3Cu^{2+}+2Al\to 2Al^{3+}+3Cu$$

(2) $$Cu^{2+}+2H_{2}O\to 2H^{+}+Cu{\left(OH\right)}_{2}$$

(3) $$6H^{+}+2Al\to 2Al^{3+}+3H_{2}$$

**Fig. 11.**
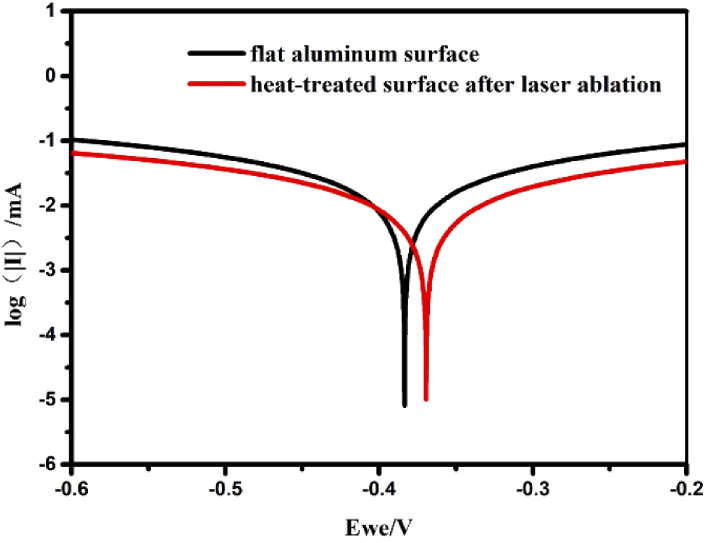
Tafel plots of the flat aluminum surface and heat-treated surface after laser ablation in 3.5 wt % NaCl solution.

Moreover, for electrochemical measurements, the corrosion potential (Ecorr) and corrosion current density (Icorr) can be obtained by the extrapolation method in this polarization (Tafel) plots. The Tafel plots are shown in Fig. 11. The corrosion voltage of the flat surface is -384.118 mV and the one on the superhydrophobic surface is -369.443 mV. As for the corrosion current, the flat surface is 22.849 μA and the superhydrophobic surface is 13.4 μA. The values of the Ecorr, Icorr, βa, and βc derived from Tafel plots showed in Table 2 [34]. Base on the Icorr, CR (corrosion rate) can be gotten on Eq. (4)

**Table 2.** Corrosion Current Density (Icorr), Corrosion Potential (Ecorr), Anodic Slope (βa) and Cathodic Slope (βc), CR and the CIE of the flat aluminum surface and heat-treated surface after laser ablation

Sample	Icorr (A cm^−2^)	Ecorr (mV)	βa (mV/ dec)	βc (mV/ dec)	CR (mm/year)	CIE (%)
Flat aluminum surface	2.2849 × 10^−5^	-384.118	311.2	307.8	0.255021	0
Heat-treated surface after laser ablation	1.34 × 10^−5^	-369.443	309.3	310.7	0.149559	41.4

(4) $$\text{CR}(\text{mm}/\text{year})=\frac{3.27\times 10^{-3}\times Icorr\times M}{nd}$$

in which M is the relative atomic mass of metal (g/mol), d is the density of the metal (g/cm^3^) and n is the number of electrons required to oxidize an atom of the element in the corrosion process. The corrosion inhibition efficiency (CIE) can be calculated by Eq. (5):

(5) $$CIE/\%=\frac{Icorr-I^{\prime }corr}{Icorr}\times 100$$

Icorr is the corrosion current of the flat aluminum surface and Icorr is the corrosion of the heat-treated surface after laser ablation [35].

The heat-treated aluminum surface showed better anti-corrosion property due to its superhydrophobicity. Additionally, the superhydrophobic aluminum surface showed a high contact angle to acid and base, as shown in Supplement 1, Fig. S5. Therefore, the superhydrophobic aluminum surface using femtosecond laser fabrication and heat treatment had the anti-corrosion property, which can prevent or slow down the corrosion reaction on its surface.

## 5. Conclusion

The environment-friendly method of fabricating nano/micro structures by the femtosecond laser ablation with a grid pattern of 100 μm step size, followed by the heat treatment post-process at 200°C for only 30 minutes can produce superhydrophobic aluminum surface in an extremely short time, without using any harsh chemical coatings. The wettability conversion time is reduced incredibly from 30 days to 30 minutes, which is shortened approximately 1440 times. All the samples demonstrated for the stable superhydrophobicity for over five months (high contact angles as 160° and small sliding angles as smaller than 5°). The presented technique demonstrated its reproductivity and feasibility of large-area fabrication, which is advantageous for manufacturing and intended industrial applications. The interaction between femtosecond laser and the metal surface can provide different surface morphology as well as different surface chemistry compared to the case of nanosecond laser, which is important for the wettability conversion from superhydrophilic to superhydrophobic. Moreover, the produced superhydrophobic aluminum surfaces exhibited brilliant properties such as repellence with various types of liquids (milk, coffee, CuPc, R6G, HCl, NaOH, and CuCl_2_), self-healing effect, and anti-corrosion. The mechanism for wettability conversion, for self-healing property, and for anti-corrosion property were also explained. The studied technique can open a new environment-friendly approach in the production of a superhydrophobic metal surface for various practical applications.

## Notes

See Supplement 1 for supporting content.

## References

[1] Jiang L. Zhao Y. Zhai J., “A lotus-leaf-like superhydrophobic surface: a porous microsphere/nanofiber composite film prepared by electrohydrodynamics,” Angew. Chem., Int. Ed. 43(33), 4338–4341 (2004). 10.1002/anie.200460333 15368387

[2] Onda T. Shibuichi S. Satoh N. Tsujii K., “Super-Water-Repellent Fractal Surfaces,” Langmuir 12(9), 2125–2127 (1996). 10.1021/la950418o

[3] Gau H. Herminghaus S. Lenz P. Lipowsky R., “Liquid morphologies on structured surfaces: from microchannels to microchips,” Science 283(5398), 46–49 (1999). 10.1126/science.283.5398.46 9872735

[4] Thieme M. Streller F. Simon F. Frenzel R. White A. J., “Superhydrophobic aluminium-based surfaces: Wetting and wear properties of different CVD-generated coating types,” Appl. Surf. Sci. 283, 1041–1050 (2013). 10.1016/j.apsusc.2013.07.065

[5] Liu H. Feng L. Zhai J. Jiang L. Zhu D., “Reversible Wettability of a Chemical Vapor Deposition Prepared ZnO Film between Superhydrophobicity and Superhydrophilicity,” Langmuir 20(14), 5659–5661 (2004). 10.1021/la036280o 16459574

[6] Li Z. Zheng Y. Zhao J. Cui L., “Wettability of Atmospheric Plasma Sprayed Fe, Ni, Cr and Their Mixture Coatings,” J. Therm. Spray Technol. 21(2), 255–262 (2012). 10.1007/s11666-011-9728-8

[7] Wang J. Li D. Liu Q. Yin X. Zhang Y. Jing X. Zhang M., “Fabrication of hydrophobic surface with hierarchical structure on Mg alloy and its corrosion resistance,” Electrochim. Acta 55(22), 6897–6906 (2010). 10.1016/j.electacta.2010.05.070

[8] Song J. Xu W. Liu X. Lu Y. Wei Z. Wu L., “Ultrafast fabrication of rough structures required by superhydrophobic surfaces on Al substrates using an immersion method,” Chem. Eng. J. 211-212, 143–152 (2012). 10.1016/j.cej.2012.09.094

[9] Malinauskas M. Zukauskas A. Hasegawa S. Hayasaki Y. Mizeikis V. Buividas R. Juodkazis S., “Ultrafast laser processing of materials: from science to industry,” Light: Sci. Appl. 5(8), e16133 (2016). 10.1038/lsa.2016.133 30167182 PMC5987357

[10] Vorobyev A. Y. Guo C., “Direct femtosecond laser surface nano/microstructuring and its applications,” Laser Photonics Rev. 7(3), 385–407 (2013). 10.1002/lpor.201200017

[11] Vorobyev A. Y. Guo C., “Metal pumps liquid uphill,” Appl. Phys. Lett. 94(22), 224102 (2009). 10.1063/1.3117237

[12] Fadeeva E. Schlie S. Koch J. Chichkov B. N. Vorobyev A. Y. Guo C. L., *Femtosecond Laser-Induced Surface Structures on Platinum and Their Effects on Surface Wettability and Fibroblast Cell Proliferation* (2009).

[13] Vorobyev A. Y. Guo C., “Multifunctional surfaces produced by femtosecond laser pulses,” J. Appl. Phys. 117(3), 033103 (2015). 10.1063/1.4905616

[14] Vorobyev A. Y. Guo C., “Femtosecond laser structuring of titanium implants,” Appl. Surf. Sci. 253(17), 7272–7280 (2007). 10.1016/j.apsusc.2007.03.006

[15] Long J. Zhong M. Zhang H. Fan P., “Superhydrophilicity to superhydrophobicity transition of picosecond laser microstructured aluminum in ambient air,” J. Colloid Interface Sci. 441, 1–9 (2015). 10.1016/j.jcis.2014.11.015 25481645

[16] Kietzig A. M. Hatzikiriakos S. G. Englezos P., “Patterned superhydrophobic metallic surfaces,” Langmuir 25(8), 4821–4827 (2009). 10.1021/la8037582 19267439

[17] Li B.-j. Li H. Huang L.-j. Ren N.-f. Kong X., “Femtosecond pulsed laser textured titanium surfaces with stable superhydrophilicity and superhydrophobicity,” Appl. Surf. Sci. 389, 585–593 (2016). 10.1016/j.apsusc.2016.07.137

[18] Crawford R. J. Ivanova E. P., *The design of superhydrophobic surfaces* (2015).

[19] Modestov A. D. Emelyanenko K. A. Emelyanenko A. M. Domantovsky A. G. Boinovich L. B., “Application of laser micro- and nanotexturing for the fabrication of superhydrophobic corrosion-resistant coatings on aluminum,” Russ. Chem. Bull. 65(11), 2607–2611 (2016). 10.1007/s11172-016-1625-3

[20] Emelyanenko K. A. Sanzharovsky N. A. Chulkova E. V. Ganne A. A. Emelyanenko A. M. Boinovich L. B., “Superhydrophobic corrosion resistant coatings for copper via IR nanosecond laser processing,” Mater. Res. Express 5(11), 115001 (2018). 10.1088/2053-1591/aadc16

[21] Ngo C. V. Chun D. M., “Fabrication of un-coated transparent superhydrophobic sapphire surface using laser surface ablation and heat treatment,” CIRP Ann. 67(1), 571–574 (2018). 10.1016/j.cirp.2018.04.085

[22] Ngo C. V. Chun D. M., “Control of laser-ablated aluminum surface wettability to superhydrophobic or superhydrophilic through simple heat treatment or water boiling post-processing,” Appl. Surf. Sci. 435, 974–982 (2018). 10.1016/j.apsusc.2017.11.185

[23] Chichkov B. N. Momma C. Nolte S. vonAlvensleben F. Tunnermann A., “Femtosecond, picosecond and nanosecond laser ablation of solids,” Appl. Phys. A: Mater. Sci. Process. 63(2), 109–115 (1996). 10.1007/BF01567637

[24] Fang R. Vorobyev A. Guo C., “Direct visualization of the complete evolution of femtosecond laser-induced surface structural dynamics of metals,” Light: Sci. Appl. 6(3), e16256 (2017). 10.1038/lsa.2016.256 30167238 PMC6062174

[25] Wasserman D., “Nanosecond modulation of thermal emission,” Light: Sci. Appl. 8(1), 68 (2019). 10.1038/s41377-019-0179-1 31645916 PMC6804789

[26] Li Q. Lao H. Lin J. Chen Y. Chen X., “Study of femtosecond ablation on aluminum film with 3D two-temperature model and experimental verifications,” Appl. Phys. A 105(1), 125–129 (2011). 10.1007/s00339-011-6579-6

[27] Glover T. E. Ackerman G. D. Lee R. W. Padmore H. A. Young D. A., “Metal–insulator transitions in an expanding metallic fluid: particle formation during femtosecond laser ablation,” Chem. Phys. 299(2-3), 171–181 (2004). 10.1016/j.chemphys.2003.11.042

[28] Wenzel R. N., “Resistance of Solid Surfaces To Wetting by Water,” Ind. Eng. Chem. 28(8), 988–994 (1936). 10.1021/ie50320a024

[29] Banerjee S., “Alumina Nanoparticles and Alumina-Based Adsorbents for Wastewater Treatment,” in *Nanomaterials for Wastewater Remediation* (Butterworth-Heinemann, 2016), pp. 239–272.

[30] Gu B. Schmitt J. Chen Z. Liang L. McCarthy J. F., “Adsorption and desorption of different organic matter fractions on iron oxide,” Geochim. Cosmochim. Acta 59(2), 219–229 (1995). 10.1016/0016-7037(94)00282-Q

[31] Takeda S. Fukawa M. Hayashi Y. Matsumoto K., “Surface OH group governing adsorption properties of metal oxide films,” Thin Solid Films 339(1-2), 220–224 (1999). 10.1016/S0040-6090(98)01152-3

[32] Wade L. G., *Organic chemistry*, Pearson Prentice Hall, Upper Saddle River, N.J. (2006).

[33] Yohe S. T. Kopechek J. A. Porter T. M. Colson Y. L. Grinstaff M. W., “Triggered Drug Release from Superhydrophobic Meshes using High-Intensity Focused Ultrasound,” Adv. Healthcare Mater. 2(9), 1204–1208 (2013). 10.1002/adhm.201200381 PMC3923359 23592698

[34] Groysman A., Corrosion for Everybody (2010).

[35] Vilaro I. Yague J. L. Borros S., “Superhydrophobic Copper Surfaces with Anticorrosion Properties Fabricated by Solventless CVD Methods,” ACS Appl. Mater. Interfaces 9(1), 1057–1065 (2017). 10.1021/acsami.6b12119 27977129

