# Supplementary material for: Rapid fabrication of anti-corrosion and self-healing superhydrophobic aluminum surfaces through environmentally friendly femtosecond laser processing
Source: Opt Express. 2020 Nov 10;28(24):35636–50. doi: 10.1364/OE.400804 (PMC7771893; doi:10.1364/OE.400804)
Supplement: Supplementary file 1 [file oe-28-24-35636-s001.pdf]

Supplemental Document

Optics EXPRESS

Rapid fabrication of anti-corrosion and self-healing superhydrophobic aluminum surfaces through environmentally friendly femtosecond laser processing: supplement

GAN YUAN,[1,2] YU LIU,[1,2] CHI-VINH NGO,[1,4] AND CHUNLEI GUO[3,5]

[1] *The Photonics Laboratory, State Key Laboratory of Applied Optics, Changchun Institute of Optics, Fine Mechanics and Physics, Chinese Academy of Sciences, Changchun, Jilin 130033, China*
[2] *University of Chinese Academy of Science, Beijing 100049, China*
[3] *The Institute of Optics, University of Rochester, Rochester, New York 14637, USA*
[4] *chivinh@ciomp.ac.cn*
[5] *guo@optics.rochester.edu*

This supplement published with The Optical Society on 10 November 2020 by The Authors under the terms of the Creative Commons Attribution 4.0 License in the format provided by the authors and unedited. Further distribution of this work must maintain attribution to the author(s) and the published article's title, journal citation, and DOI.

Supplement DOI: https://doi.org/10.6084/m9.figshare.13058372

Parent Article DOI: https://doi.org/10.1364/OE.400804

supplemental document

Table S1 of contact angle and sliding angle of the sample by different step size

Step size (μm)	0 month		2 month	
	CA	SA	CA	SA
100	158.9° ± 0.9°	2.4° ± 0.6°	159.6° ± 0.4°	2.5° ± 0.6°
300	157.9° ± 2.3°	6.1° ± 1.0°	154.9° ± 1.2°	9.2° ± 2.1°
500	155.8° ± 2.6°	20.8° ± 2.8°	151.7° ± 1.1°	27.5° ± 7.4°

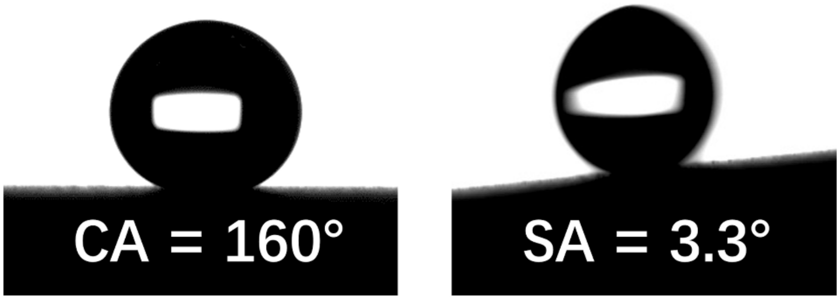

Figure S1 The contact angle and the sliding angle of the heat-treated surface after laser ablation surface with 20 mm * 20 mm

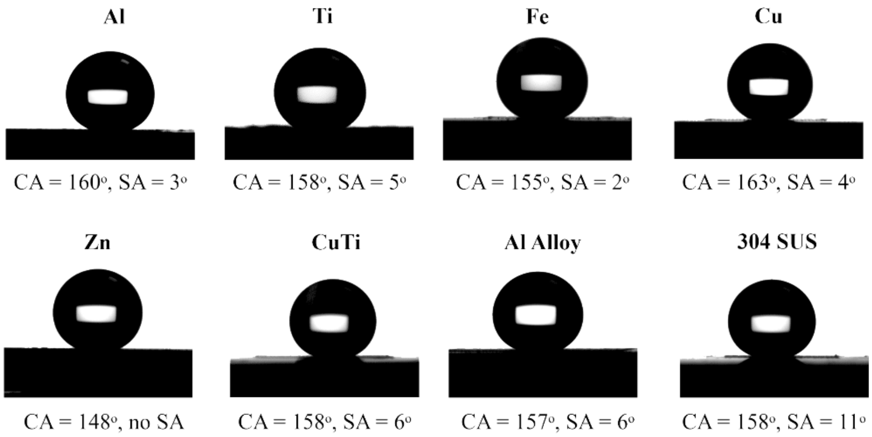

Figure S2 30 minutes of heat treatment after femtosecond laser ablation on various common metals and metal alloys (Al, Ti, Fe, Cu, Zn, CuTi, Al alloy and 304 stainless steel)

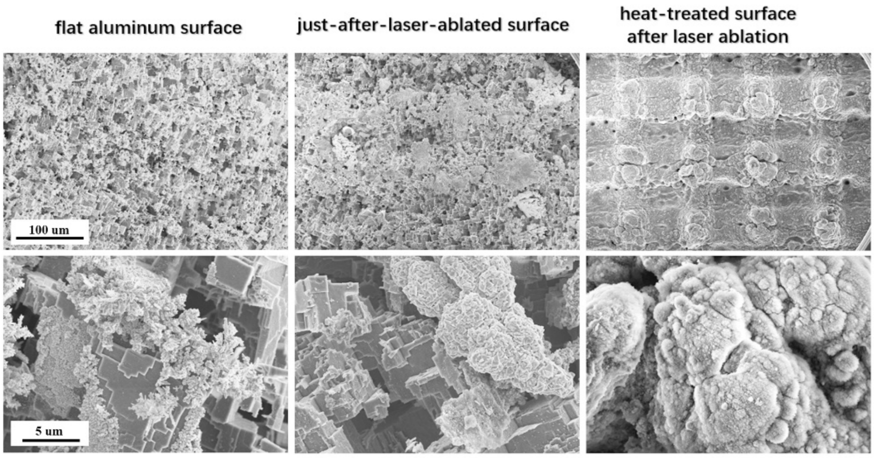

Figure S3 The SEM results of the flat aluminum surface, just-after-laser-ablated surface and heat-treated surface after laser ablation after putting in $CuCl_2$ for 3 minutes

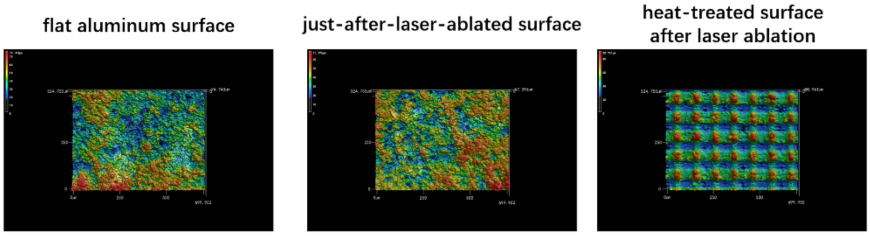

Figure S4 The 3D structure results of flat aluminum surface and just-after-laser-ablated surface, the heat treated surface after laser ablation after putting in $CuCl_2$ for 3 minutes.

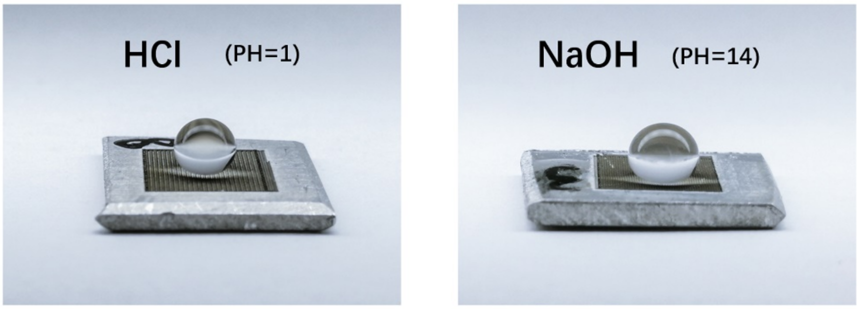

Figure S5 High contact angle of superhydrophobic aluminum surface to hydrochloric acid (HCl with pH=1) and sodium hydroxide (NaOH with pH=14)
